# Targeted Mutagenesis in Atlantic Salmon (*Salmo salar* L.) Using the CRISPR/Cas9 System Induces Complete Knockout Individuals in the F0 Generation

**DOI:** 10.1371/journal.pone.0108622

**Published:** 2014-09-25

**Authors:** Rolf B. Edvardsen, Sven Leininger, Lene Kleppe, Kai Ove Skaftnesmo, Anna Wargelius

**Affiliations:** Institute of Marine Research, Bergen, Norway; Rutgers University -New Jersey Medical School, United States of America

## Abstract

Understanding the biological function behind key proteins is of great concern in Atlantic salmon, both due to a high commercial importance and an interesting life history. Until recently, functional studies in salmonids appeared to be difficult. However, the recent discovery of targeted mutagenesis using the CRISPR/Cas9 (clustered regularly interspaced palindromic repeats/CRISPR-associated) system enables performing functional studies in Atlantic salmon to a great extent. We used the CRISPR/Cas9 system to target two genes involved in pigmentation, *tyrosinase* (*tyr*) and *solute carrier family 45, member 2* (*slc45a2*). Embryos were assayed for mutation rates at the 17 somite stage, where 40 and 22% of all injected embryos showed a high degree of mutation induction for *slc45a2* and *tyr*, respectively. At hatching this mutation frequency was also visible for both targeted genes, displaying a graded phenotype ranging from complete lack of pigmentation to partial loss and normal pigmentation. CRISPR*slc45a2*/Cas9 injected embryos showing a complete lack of pigmentation or just a few spots of pigments also lacked wild type sequences when assaying more than 80 (*slc45a2*) sequence clones from whole embryos. This indicates that CRISPR/Cas9 can induce double-allelic knockout in the F0 generation. However, types and frequency of indels might affect the phenotype. Therefore, the variation of indels was assayed in the graded pigmentation phenotypes produced by CRISPR/Cas9-*slc45a2*. The results show a tendency for fewer types of indels formed in juveniles completely lacking pigmentation compared to juveniles displaying partial pigmentation. Another interesting observation was a high degree of the same indel type in different juveniles. This study shows for the first time successful use of the CRISPR/Cas9 technology in a marine cold water species. Targeted double-allelic mutations were obtained and, though the level of mosaicism has to be considered, we demonstrate that F0 fish can be used for functional studies in Atlantic salmon.

## Introduction

Atlantic salmon is an important commercial aquaculture species. Likewise, the growing salmon industry displays a demand for healthy, fast growing and tasty fish. To improve knowledge and to solve bottlenecks of salmon aquaculture, the salmon genome is currently being sequenced [1]. To further implement findings gained from the salmon genome, functional studies of genes linked to health, growth, welfare and filet quality will be necessary. This will increase our knowledge and possibly explain causative relations to some of the challenges present in the aquaculture industry such as egg/sperm quality, reproductive mechanisms, growth rate, disease resistance and immunological mechanisms. In the era of genome wide association studies many genes will be directly linked to a trait and further functional characterization of these genes will significantly improve selective breeding of associated traits.

Functional studies in salmon embryos have so far been difficult to implement, but a few studies have employed knockdown techniques in salmonids using morpholinos [2]–[4]. However, current approaches have not proven to be very efficient. This can partly be ascribed to the characteristics of the egg being difficult to precisely inject due to its large and opaque features and almost invisible first cell stage [5]. This together with the variable efficiencies using morpholinos makes it even more difficult in this non-model species [6].

In recent years the ability to specifically create targeted mutations by gene editing techniques has steadily improved. The first gene editing experiments in teleosts were conducted in zebrafish, starting with targeted mutagenesis using zinc finger nucleases (ZFN, [7], [8]) followed by the more efficient transcription activator-like effectors (TALENS, [9]). A study in rainbow trout used ZFN to induce targeted mutagenesis in the *sdy* gene, with the purpose to confirm its function in sex determination [10]. The ZFN technology has also been applied to a catfish species, with the aim to elucidate muscle growth mechanisms [11]. The problem using this methodology in salmonids is their long generation time (2–4 years) as previously used targeted mutagenesis protocols do not efficiently induce bi-allelic mutations and therefore require breeding for two more generations to induce a homozygous mutation in targeted genes.

Recently, with the CRISPR (clustered regularly interspaced palindromic repeats)/Cas9 (CRISPR-associated) system, a highly efficient technology has been developed, which has shown to specifically induce mutations in many model and non-model animals including zebrafish and tilapia [12], [13]. This technology is so efficient in zebrafish and *Xenopus* that it can induce gene specific bi-allelic mutations in the first (F0) generation [14], [15]. This feature makes this new technology very promising for use in non-model organisms with longer life spans such as the Atlantic salmon.

The CRISPR/Cas9 system was identified in bacteria and archaea, serving as an immune system against bacteriophages and foreign DNA [16]–[19]. In these organisms the CRISPR loci incorporates foreign DNA, this DNA is then synthesized into short CRISPR RNAs (crRNA) which bind to a transactivating RNA (tracrRNA) and to foreign DNA catalyzing cleavage and destruction by recruiting the RNA-guided DNA nuclease Cas9 [20]. A construct which has been tailored for targeted mutations is a fusion of crRNA and tracrRNA functioning as a guide RNA (gRNA) which in combination with the Cas9 nuclease can then induce double DNA strand breaks [21]. The mutation is introduced when the doublestrand break is repaired by either high-fidelity homologous recombination or error prone non-homologous end-joining (NHEJ).

The aim of the present study was to attempt creating specifically induced bi-allelic mutations in target genes in Atlantic salmon using the CRISPR/Cas9 system. For this purpose we selected genes producing pronounced pigmentation phenotypes in zebrafish enabling a direct visual enumeration [22]–[23]. Specific target oligonucleotides corresponding to two genes (*tyrosinase* and *slc45a2*) known to be involved in pigmentation in zebrafish and other species were cloned into a CRISPR vector developed for zebrafish [14]. *In vitro* transcribed RNAs from the cloned constructs were injected into one-cell stage Atlantic salmon embryos together with Cas9 mRNA. Mutation rates were measured in whole embryos prior to hatch and in DNA prepared from finclip versus whole juvenile fish showing lack of pigmentation. Here, we present the first targeted genome editing employing the CRISPR/Cas9 technology on salmonid fish and further extend the list of successfully tested non-model species where functional studies and induction of mutations have previously been problematic.

## Materials and Methods

### Cloning of target sites for gRNAs

Using a draft salmon genome sequence (AGKD00000000.1) both *tyrosinase* (*tyr*) and *slc45a2* exon sequences were predicted by comparing to published sequences in salmon for *tyr* (Accession: NM_001123643) and in zebrafish for *slc45a2* (NP_001103847). From the predicted sequences in the salmon genome the *slc45a2* gene was found split into two contigs (Accession: AGKD01080285, AGKD01048537). The salmon *tyrosinase* (*tyr*) coding sequence was found split into four genomic contigs (Accession: AGKD01095509, AGKD01379509, AGKD01094529, AGKD01346345). Suitable oligonucleotides for CRISPR targeting were selected using an online tool provided by http://zifit.partners.org/ZiFiT/ [24]. Candidate target sequences were compared to the current salmon genome draft to exclude unwanted off-target cleavage. Target site and oligonucleotide sequences for salmon *slc45a2* and *tyr* are listed in Table S1. Oligonucletides were annealed and cloned into pT7-gRNA [14], Addgene ID# 46759 as described below: 2 µM of each forward and reverse oligonucleotide was annealed in annealing buffer (0.4 M Tris pH 8, 0.2 M MgCl_2_, 0.5 M NaCl, 10 mM EDTA pH 8) by incubating at 95°C for 5 min, followed by ramping down to 4°C at −2°C/min. 3 µl of annealed oligonucleotides were ligated into 5 ng of BsmBI, BglII, SalI digested pT7-gRNA and gel-extracted using Quick T4 DNA Ligase (NEB) and subsequently cloned using XL1-blue competent cells (Stratagene). Plasmids were prepared using a QIAprep Spin Miniprep Kit (Qiagen).

### *In-vitro* transcription of gRNA and Cas9 mRNA

pT7-gRNA was linearized using BamHI-HF (NEB), containing the respective cloned target sites for either *slc45a2* or *tyr*, were prepared using a QIAprep column (Qiagen) and transcribed using the MEGAscript T7 kit (Ambion) according to the manufacturer’s protocol. The *mir*Vana miRNA Isoltation Kit was used to purify gRNAs.

For producing Cas9 nuclease mRNA, we used the pTST3-nCas9n vector optimized for Zebrafish [14] (Addgene ID# 46757). Prior to *in-vitro* transcription, the plasmid was linearized using XbaI (NEB) and cleaned up via a QIAprep Spin column. Cas9 mRNA was produced using the mMessage mMachine T3 kit (Ambion) and purified using an RNeasy MiniKit spin column (Qiagen).

### Injection procedures

Salmon eggs and sperm obtained from Aquagen (Trondheim, Norway) were sent overnight to Matre Aquaculture station at IMR. Eggs were subsequently fertilized with sperm in fresh water (6–8°C) containing 0.5 mM reduced Gluthathione as described earlier for rainbow trout [25]. After fertilization, embryos were incubated 2–3 hours at 6–8°C until the first cell was visible. Eggs were subsequently injected with a mix containing 50 ng/µl gRNA and 150 ng/µl Cas9 mRNA in MilliQ H_2_O using the picospritzer III (Parker Automation, UK) and needles from Narishige (Japan). After injection, eggs were incubated at 6°C until hatching.

### Analysis of mutations

DNA was obtained from embryos, juveniles and fin clips using DNeasy Blood & Tissue kit (Qiagen) or AllPrep DNA/RNA kit (Qiagen) with the following modifications: Eggs were squashed in Buffer ATL and Proteinase K (DNeasy Blood & Tissue kit, Qiagen), incubated at 56°C for 2–4 h and subjected to phenol/chloroform extraction prior to DNA purification. Briefly, 1 vol. of UltraPure Phenol/Chloroform/Isoamylalcohol (25∶24∶1, Lifetechnologies) was added, mixed and phases separated by centrifugation. The upper phase was mixed with 1 vol. of chloroform, centrifuged and 1 vol. of Buffer AL and 100% EtOH (DNeasy Blood & Tissue kit, Qiagen) was added to the DNA containing aqueous phase and subjected to subsequent steps of the DNeasy Blood & Tissue kit protocol. Juveniles (separated from the yolk sac) and fin clips were homogenized using Zirconium oxide beads and a homogenizer (Precellys) in buffer ATL or buffer RLTplus/β-mercaptoethanol (AllPrep kit, Qiagen) prior to DNA extraction. PCR was performed on genomic DNA to obtain a fragment that covered the targeted mutagenesis site (PCR primer sequences are listed in Table S1). Fragments were both directly sequenced and subcloned into pCR4-TOPO using the TOPO TA cloning kit for sequencing (Invitrogen) to either measure the general effect in the target site in the whole preparation or in single sequences from clones to assess the level of mosaic mutation rate in each individual or sample.

### Ethics statement

All animal experiments within the study were approved by NARA, the governmental Norwegian Animal Research Authority (http://www.fdu.no/fdu/). To avoid unnecessary pain all fish larvae were sedated with metomidate prior to sampling.

## Results and Discussion

For the establishment of the CRISPR/Cas method in salmon, target genes were selected based on their known role in pigmentation of fish, as a resulting phenotype is clearly visible at early developmental stages and throughout life. Since salmonid genomes are partly tetraploid [26], the two selected pigmentation genes, *tyrosinase* and *slc45a2*, were also chosen because both occur only once in the genome making them suitable for gene knockout. Tyrosinase (*tyr*) is essential for normal pigmentation both in medaka [27]–[28] and zebrafish [22]. In addition Tyr has previously been shown to affect pigmentation in rainbow trout using morpholinos [29]. Likewise, Slc45a2 is essential for pigmentation in both zebrafish [23] and medaka [30].

**Figure 1:**
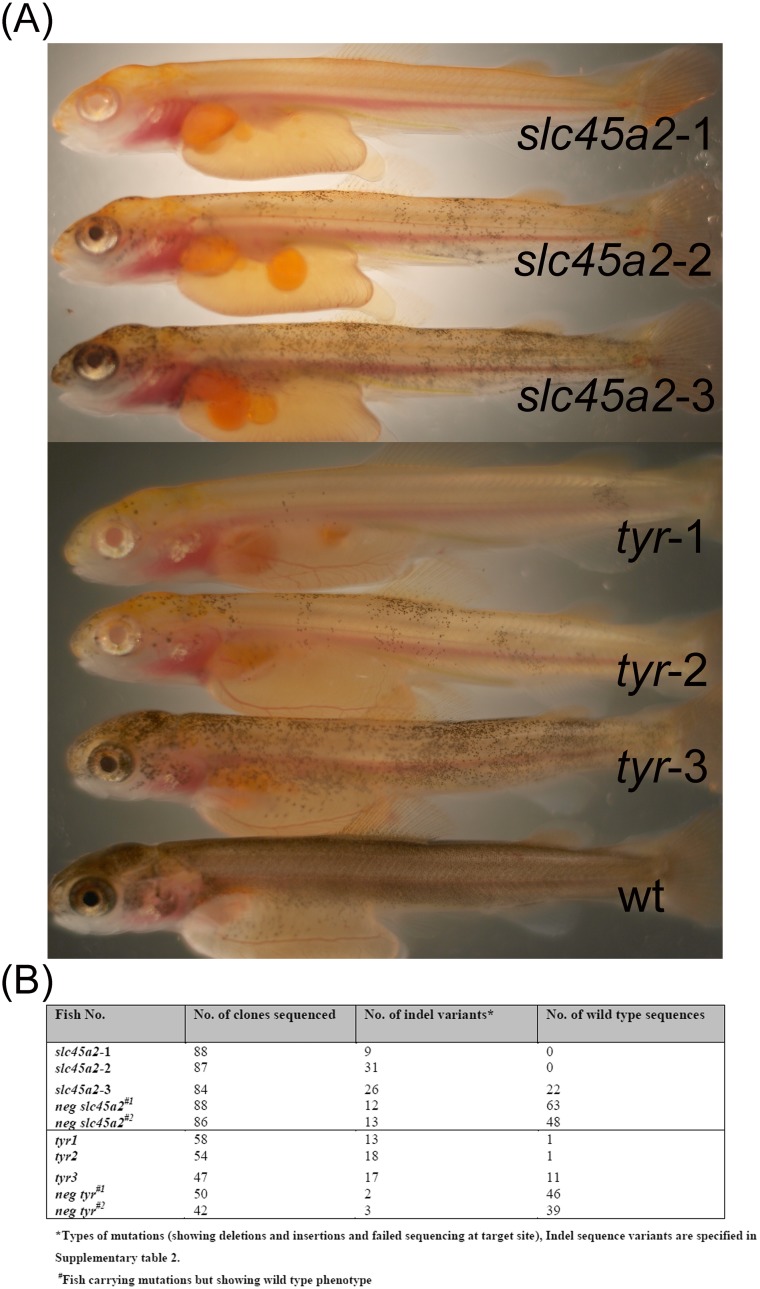
Graded levels of phenotypes induced by CRISPR*slc45a2*/Cas9 (*slc45a2-*1 to *slc45a2-*3) and CRISPR*tyr*/Cas9 (*tyr*-1 to *tyr*-3) found in picture A. Mutation rates and number of indel variants found in Atlantic salmon whole juvenile fish extracts at 650 day° (B).

**Figure 2:**
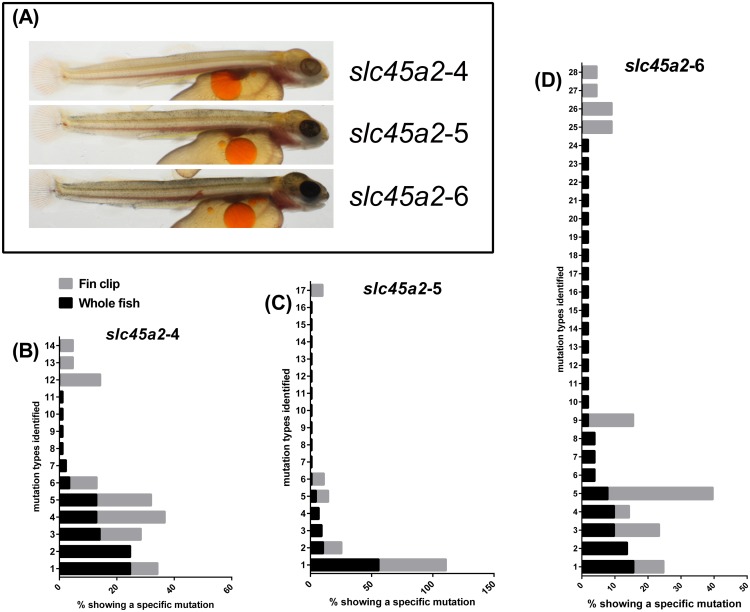
Level of indel abundances in fin clip versus whole juvenile DNA preparation from CRISPR*slc45a2*/Cas9 (*slc45a2-*4 to *slc45a2-*6) at 600 day°. (A) Fish analyzed for indels in fin clip and whole juvenile fish preparations. (B–D) Frequency [%] (x-axis) of a specific indel (y-axis) in fin clip (grey bars) and whole fish (black bars) of Atlantic salmon juveniles at 600 day°.

Since development is relatively slow in Atlantic salmon, it takes almost three months until a visible pigmentation phenotype can be observed in hatched embryos at about 500 day° (∼84 days at 6°C [5]). In salmon, the developmental stage is calculated as day° by multiplying the incubation temperature (in °C) with the amount of days since fertilization. At stages prior to visual examination, embryos can be screened by PCR for targeted mutations. Consequently, injected embryos were harvested two weeks after fertilization (17 somite stage, 72 day°), DNA was extracted from whole embryos and PCR screenings were performed for both gRNA targets, CRISPR*slc45a2*/Cas9 (n = 20 embryos) and CRISPR*tyr*/Cas9 (n = 9 embryos). This analysis revealed that both CRISPR*slc45a2*/Cas9 and CRISPR*tyr*/Cas9 resulted in induced mutations in 40% and 22% of the embryos at early developmental stages, respectively (see Table 1). This reflects that in our experiments the overall mutation rate is lower than what has been observed in zebrafish (75–90%) [14] but within a similar range as it has been obtained for tilapia (24–50%) [13]. A possible reason for a lower mutation rate in Atlantic salmon can be the lower incubation temperature (6–8°C) causing the Cas9 enzyme to be less effective. Another more plausible explanation is the injection procedure. Because of the opaque character of newly fertilized salmon eggs and the hardness of the chorion it is difficult to inject directly into the cytoplasm of the first cell. Therefore, the relatively low number of embryos displaying mutations is possible due to failed injections. However, we assume that successful injections result in a high incidence of mutations (Figure 1 and 2) which implies that the mutation efficiency is as high as previously observed for zebrafish [14]. An increased early mutation frequency could possibly also be obtained by injecting the Cas9 protein directly or by improving the timing of injection after fertilization. It has been shown that the introduction of off-target mutations might constitute a problem using the CRISPR/Cas9 system. In this study we did not measure off-target mutations, however previous studies in zebrafish has shown a low off-target mutation frequency produced by CRISPR [31]. CRISPR sites were also carefully selected in this study to prevent off-target effects.

**Table 1:** Mutation rate in CRISPR/Cas9 embryos and juveniles.

Targeted Gene	17 somite stageembryos (72 day°)	Phenotype 650 day°	Mutants withoutphenotype 650 day°
*slc45a2*	40% (8/20)	8.7 % (16/184)	9.5% (2/21)
*tyr*	22 % (2/9)	5.1% (19/371)	45% (5/11)

At 650 day° phenotypes in mutated juvenile groups were visually determined and 8.7% and 5.1% of juveniles displayed a graded loss of pigmentation (Figure 1A). The similar graded degree of phenotype induction has previously been observed in both zebrafish and *Xenopus* for targeting the *tyr* gene in these species [14], [32]. In contrast to these studies a dose dependent severety of phenotype could not be established, since we only used one empirically determined concentration of gRNA and Cas9 mRNA in our work. However, we cannot exclude a dose-dependent effect due to injection procedures as explained above. Fish showing a graded phenotype due to mutations in either *slc45a2* or *tyr*, were selected based on the level of pigmentation loss. Hence, the following juveniles were subjected to PCR screening and sequencing of induced mutations; full (*slc45a2*-1), almost full (*tyr*-1), medium (*slc45a2*-2, *tyr*-2) and little pigmentation loss (*slc45a2*-3, *tyr*-3, Figure 1, Table S2a–c and S3a–c). Additionally, two fish from each group, targeted for either *slc45a2* or *tyr*, which showed no visual pigmentation loss were analyzed (*neg slc45a2* ^#1^, *neg slc45a2* ^#2^, *neg tyr* ^#1^ and *neg tyr* ^#2^, Figure 1, Table S2e–f and S3e–f). To quantify mutation frequencies in each juvenile fish, DNA was extracted from the whole fish, a PCR product covering the CRISPR target site was amplified and the product was subsequently subcloned (50–100 clones per fish). In the mutated *slc45a2*-1 fish, which displayed a complete loss of pigmentation, no wild type sequences could be detected in any of the clones assayed (n = 88). However, medium pigmentation loss phenotype in *slc45a2*-2 (Figure 1, Table S2b) mutated fish also lacked wild type sequences even though 87 clones were sequenced. Simply undersampling might be an explanation for this. Nevertheless, more wild type sequences have been detected in fish with less severe phenotypes, while none or few could be found in strong (no/medium pigmentation) phenotypes. This trend was also observed in two of the five *tyr* phenotypes (*tyr*-1 and *tyr*-2), where only one wild type sequence of 58 and 54 sequenced clones, respectively, could be detected in each fish (Figure 1, Table S3a–b). Additionally, we screened 21 and 11 fish targeted for either *slc45a2* or *tyr*, displaying a wild type phenotype at 650 day° (neg *slc45a2* ^#1^, neg *slc45a2* ^#2^, *neg tyr* ^#1^ and *neg tyr* ^#2^, Figure 1B). In these groups, 9,5% (*slc45a2*) and 45% (*tyr*) of juveniles were shown to have indel mutations (Table 1, Table S2d–e and S3d–e). As expected these fish displayed a low frequency of targeted mutations explaining the wild type character of the fish (Figure 1B).

We further wanted to know if embryo/juvenile stages could be sorted based on mutation frequencies in fin clips only, instead of sacrificing the whole fish. This is important in future studies when targeting genes with unknown function and where mutated embryos/juveniles show no visual phenotype in F0. To investigate if fin clips could mirror the mutation type found in whole fish, we screened fin clip and whole fish DNA preparations from additional juveniles (600 day°) which displayed graded pigmentation phenotypes in the *slc45a2* knockout attempts. This included three fish *slc45a2*-4 which only displayed a few pigmentation spots, *slc45a2*-5 displayed some more pigmentation spots and *slc45a2*-6 displayed even more pigmentation spots (Figure 2A). These fish were screened for indel types and frequencies which are presented in Figure 2 B–D and in Table S4a–c. This comparison revealed similar frequencies for the most common indel variants in all three fish. However, among the less frequently occurring indels we observe more indel variants in the whole fish compared to fin clip. These results show that the fin clip to a certain degree mirrors the mosaicism in the knockout fish and can therefore be used as a tool to evaluate knockout phenotypes.

Another interesting finding was the independent generation of the same mutation types in different fish injected with the same gRNA (Table 2). By thorough screening of the 6 *scl45a2* mutated fish (*slc45a2*-1 to *slc45a2*-6, Figure 1A and 2A), we found 7 of the indel variants to be present in at least three of the seven fish (Table 2). However, we were not able to link the abundance of certain indel types to specific phenotypes. These mutations were all in proximity of the PAM site (bold and underlined in Table 2), which is similar to what has been observed previously in zebrafish [14], [24]. A previous study using the CRISPR/Cas9 technology in *Xenopus* also indicated that the same mutations were formed using the same construct in different F0 individuals [15]. This further suggests that individual F0 fish may hold the exact same indel variants and crosses of these can produce a non mosaic homozygous mutant already in the F1 generation. This technology is therefore especially relevant for species with long generation times like Atlantic salmon.

**Table 2:** Mutations occurring in 3 or more of the 6 CRISPR*slc45a2*/Cas9 injected fish (Figure 1 and 2).

*	Common indels formed in embryos injectedwith CRISPR/Cas9 against *slc45a2*	*slc45a2*-1	*slc45a2*-2	*slc45a2*-3	*slc45a2*-4	*slc45a2*-5	*slc45a2*-6
A	TGTTTGGTCTGGGCA**CCA**GT----------------CGGCCTGTTCCCC		17	12	23	10	10
B	TGTTTGGT--------------------------------CTGTTCCCC				1	2	2
C	TGTTTG––––––––––––––––GCCTGTTCCCC		14		15	1	1
D	TGTTTGGTCTGGGCA--------------------------------CC	7	3	7		10	10
E	TGTTTGGTCTGGGCA**CCA**GTC-----------TTATCGGCCTGTTCCCC		2			11	11
F	TGTTTGGTCTGGGCA**CCA**GAC---------------CGGCCTGTTCCCC		1			1	1
G	TGTTTGGTCTGGGCA**CCA**GT-------------TATCGGCCTGTTCCCC	18	4	2	1	4	4
H	TGTTTGGTCTGGGCACC--------------------GGCCTGTTCCCC	17		2		1	6

*Indel types A–H refer to ID shared in tables S2, S3 and S4.

In conclusion, we show here for the first time successful targeted mutagenesis in salmon using the CRISPR/Cas9 technology. We anticipate that this technology will be an important tool for functional studies in this biologically and commercially interesting fish species. In the future the use of this technology will be facilitated since the rainbow trout genome has recently been sequenced [26] and the Atlantic salmon genome has been made available (Acc. No. AGKD00000000.3). In our hands, genome editing using CRISPR/Cas9 in salmon seems to be somewhat less efficient than reported for other fish species [12]–[14], probably due to the features of the egg and hence obstacles during injections procedures. Nevertheless, using knockout of pigmentation genes as shown here, can be a useful marker in co-injections with gRNAs targeting other genes of interest which show no obvious phenotype. This approach could facilitate the selection of putative positive fish for confirming a successful knockout of targeted genes. Previous targeted knock out technologies such as TALENs and ZFN did induce lower mutation frequencies in the F0 compared to CRISPR/Cas9 [7]–[9]. Hence, due to the general high efficiency and biallelic mutation capacity of the CRISPR/Cas9 technology, the F0 generation can already be used for the evaluation of protein functions in salmonids, as has been previously demonstrated for tilapia and *Xenopus* [13], [15]. Furthermore, this paper substantiates that the stronger the knockout phenotype found in the F0 generation is, the less indel variants are found. We also tested and confirmed that fin clips can be used as an indicator of an efficient knockout. To conclude, the establishment of functional studies through the CRISPR/Cas9 technique in salmonids opens up new doors to understand their basic biology and provides a useful tool for investigating traits important for the aquaculture industry.

## Supporting Information

Table S1 **CRISPR target site oligonucleotides and PCR primers.**
(DOC)

Table S2 **Indel types found in slc45a2 fish presented in** **Figure 1** **.**
(DOC)

Table S3 **Indel types found in tyr fish presented in** **Figure 1** **.**
(DOC)

Table S4 **Indel types found in slc45a2 fish presented in** **Figure 2** **.**
(DOC)
