# Supplementary material for: Targeted Mutagenesis in Atlantic Salmon (Salmo salar L.) Using the CRISPR/Cas9 System Induces Complete Knockout Individuals in the F0 Generation
Source: PLoS One. 2014 Sep 25;9(9):e108622. doi: 10.1371/journal.pone.0108622 (PMC4177897; doi:10.1371/journal.pone.0108622)
Supplement: Table S1 — CRISPR target site oligonucleotides and PCR primers. (DOC) [file pone.0108622.s001.doc]

**Supplementary Table S1**

**CRISPR target sequence and Oligonucleotides**

| name | sequence |
| --- | --- |
| *slc45a2target*  *slc45a2F* | 5'-GGGGAACAGGCCGATAAGACTGG-3'  5’-TAGGGGAACAGGCCGATAAGAC-3’ |
| *slc45a2R*  *tyr target* | 5’-AAACGTCTTATCGGCCTGTTCC-3’  5'-GGGGGACGGGTCGGCATGTGGGG-3' |
| *tyr 1F* | 5’-TAGGGGGACGGGTCGGCATGTG-3’ |
| *tyr 1R* | 5’-AAACCACATGCCGACCCGTCCC-3’ |

PAM site underlined

**PCR Primers**

| name | sequence |
| --- | --- |
| *slc45a2 PCRF* | 5’-TGCCACAGCCTCAGAATGTACA-3’ |
| *slc45a2PCRR* | 5’-CAGATGTCCAGAGGCTGCTGCT-3’ |
| *tyr PCRF* | 5’-TGACCCGGCTGGACCTGCTGTG-3’ |
| *tyr PCRR* | 5’-ACGTAGTCAGCGGTGACGGTGT-3’ |
