# Supplementary material for: Targeted Mutagenesis in Atlantic Salmon (Salmo salar L.) Using the CRISPR/Cas9 System Induces Complete Knockout Individuals in the F0 Generation
Source: PLoS One. 2014 Sep 25;9(9):e108622. doi: 10.1371/journal.pone.0108622 (PMC4177897; doi:10.1371/journal.pone.0108622)
Supplement: Table S2 — Indel types found in slc45a2 fish presented in Figure 1 . (DOC) [file pone.0108622.s002.doc]

**Supplementary table S2.** Indels in CRISP*slc45a2*/Cas9 injected embryos. “ID shared” defines shared indel types for all 5 tested fish.

Suppl. table 2a: Type and frequency of mutations in the *slc45a2*-1 fish (see Figure 1)

| **ID shared** | **#** | **Sequence** | **frequency** |
| --- | --- | --- | --- |
|  | 1 | CTTTGTGTTTGGTCTGGGCACC---------------------------AGCATTATCACCACCC | 17x |
| A | 2 | CTTTGTGTTTGGTCTGGGCACC---------------------------AACATTATCACCACCC | 7x |
| B | 3 | CTTTGTGTTCGG---------------------------CCTGTTCCCCAACATTATCACCACCC | 6x |
| C | 4 | CTTTGTGTTTGGTCTGGGCACC---------------GGCCTGTTCCCCAACATTATCACCACCC | 17x |
| D | 5 | CTTCGTGTTTGGTCTGGGCACCAGT-----------CGGCCTGTTCCCCAACATTATCACCACCC | 7x |
|  | 6 | CTTTGTGTTTGGTCTGGGCACCAGT--------TATCGGCCTGTCCCCCAACATTATCACCACCC | 2x |
| E | 7 | CTTTGTGTTTGGTCTGGGCACCAGT--------TATCGGCCTGTTCCCCAACATTATCACCACCC | 18x |
|  | 8 | CTTTGTGTTTGGTCTGGGCACCAGTCggccagTTATCGGCCTGTTCCCCAACATTATCACCACCC | 9x |
|  | 9 | CTTTGTGTTTGGTCTGGGCACCAcaC--ccaaTTATCGGCCTGTTCCCCAACATTATCACCACCC | 5x |
|  |  | CTTTGTGTTTGGTCTGGGCACCAGTC------TTATCGGCCTGTTCCCCAACATTATCACCACCC | 0x, wild-type |
|  |  |  | 88 clones |

Target site

PAM

x insertion

X substitution

Suppl. table 2b: Type and frequency of mutations in the *slc45a2*-2 fish (see Figure 1)

| **ID shared** | **#** | **Sequence** | **frequency** |
| --- | --- | --- | --- |
|  | 1 | CTTTGTGTTT----------------------------------------------------------------------ATCGGCCTGTTCCCCAACATTATCACCACCC | 1x |
|  | 2 | CTTTGTGTT-----------------------------------------------------------------------ATCGGCCTGTTCCCCAACATTATCACCACCC | 1x |
|  | 3 | CTTTGTGTTTTGGT------------------------------------------------------------------ATCGGCCTGTTCCCCAACATTATCACCACCC | 1x |
| B | 4 | CTTTGTGTTTGGCC-------------------------------------------------------------------------TGTTCCCCAACATTATCACCACCC | 14x |
|  | 5 | CTTTGTGTTTGGTCTGGGT-------------------------------------------------------------------CTGTTCCCCAACATTATCACCACCC | 1x |
|  | 6 | CTTTGTGTTTGGTCTGGGCA-------------------------------------------------------------------------CCAACATTAACACCACCC | 3x |
|  | 7 | CTTTGTGTTTGGTCTGGGTT------------------------------------------------------------ATCGGCCTGTTCCCCAACATTATCACCACCC | 1x |
|  | 8 | CTTTGTGTTTGGTCTGGGTG------------------------------------------------------------GTGATAATGTTCCCCAACATTATCACCACCC | 1x |
|  | 9 | CTTTGTGTTTGGTCTGGGCAA-----------------------------------------------------------ATCGGCCTGTTCCCCAACATTATCACCACCC | 1x |
|  | 10 | CTTTGTGTTTGGTCTGGGCAC----------------------------------------------------------------------------CATTATCACCACCC | 1x |
|  | 11 | CTTTGTGTTTGGTCTGGGCACCA----------------------------------------------------------------TGTTCCCCAACATTATCACCACCC | 1x |
|  | 12 | CTTTGTGTTTGGTCTGGGCACCG----------------------------------------------------------------TGTTCCCCAACATTATCACCACCC | 2x |
|  | 13 | CTTTGTGTTTGGTCTGGGCACCA-G-------------------------------------------------------ACCGGCCTGTTCCCCAACATTATCACCACCC | 1x |
|  | 14 | CTTTGTGTTTGGTCTGGGCACCT-G--------------------------------------------------------TCGGCCTGTTCCCCAACATTATCACCACCC | 1x |
|  | 15 | CTTTGTGTTTGGTCTGGGCACCA------------------------------------------------------------GGCCTGTTCCCCAACATTATCACCACCC | 1x |
| F | 16 | CTTTGTGTTTGGTCTGGGCACCA-G-------------------------------------------------------A-CGGCCTGTTCCCCAACATTATCACCACCC | 3x |
|  | 17 | CTTTGTGTTTGGTCTGGGCACCA-G--------------------------------------------------------TCGGCCTGGTGCCCAACATTATCACCACCC | 7x |
|  | 18 | CTTTGTGTTTGGTCTGGGCACCA-G--------------------------------------------------------TCGACCTGTTCCCCAACATTATCACCACCC | 1x |
| D | 19 | CTTTGTGTTTGGTCTGGGCACCA-G--------------------------------------------------------TCGGCCTGTTCCCCAACATTATCACCACCC | 17x |
|  | 20 | CTTTGTGTTTGGTCTGGGCACCA-------------------------------------------------------TTATCGGCCTGTTCCCCAACATTATCACCACCC | 3x |
| E | 21 | CTTTGTGTTTGGTCTGGGCACCA-G-----------------------------------------------------TTATCGGCCTGTTCCCCAACATTATCACCACCC | 4x |
| G | 22 | CTTTGTGTTTGGTCTGGGCACCA-G------------------------------------------------------TATCGGCCTGTTCCCCAACATTATCACCACCC | 1x |
|  | 23 | CTTTGTGTTTGGTCTGGGCACCA-G-----------------------------------------------------TTATCAGCCTGTTCCCCAACATTATCACCACCC | 1x |
|  | 24 | CTTTGTGTTTGGTCTGGGCACC--G-----------------------------------------------------TTATCGGCCTGTTCCCCAACATTATCACCACCC | 4x |
|  | 25 | CTTTGTGTTTGGTCTGGGCACCAaacacaaag------------------------------------------------GTCGGCCTGTTCTCCAACATTATCACCACCC | 1x |
|  | 26 | CTTTGTGTTTGGTCTGGGCACCAaacacaaag------------------------------------------------GTCGGCCTGTTCCCCAACATTATCACCACCC | 2x |
|  | 27 | CTTTGTGTTTGGTCTGGGCACCCCACtccccatgagca----------------------------------------TTATCGGCCTGTTCCCCAACATTATCACCACCC | 1x |
|  | 28 | CTTTGTGTTTGGTCTGGGCACCAGTCgttcgtg------------------------------------caccagtcgTTATCGGCCTGTTCCCCAACATTATCACCGCCC | 3x |
|  | 29 | CTTTGTGTTTGGTCTGGGCACCAGTCagaagcagctctagtgatgcccgtgggtccactctgtcattatctcactacaTTATCGGCCTGTTCCCCAACATTATCACCACCC | 1x |
|  | 30 | CTTTGTGTTTGGTCTGGGCACCAGTCagaagcagctctagtgatgcacgtgggtccactctgtcattatctcactgtaTTATCGGCCTGTTCCCCAACATTATCACCACCC | 2x |
|  | 31 | CTTTGTGTTTGGTCTGGGCACCAGTC----------------------------------------------------TTATCGGCCTGTTCCCCAGCATTATCACCACCC | 2x |
|  |  | CTTTGTGTTTGGTCTGGGCACCAGTC----------------------------------------------------TTATCGGCCTGTTCCCCAACATTATCACCACCC | 3x, wild-type |
|  |  |  | 87 clones |

Suppl. table 2c: Type and frequency of mutations in the *slc45a2*-3 fish (see Figure 1).

| **ID shared** | **#** | **Sequence** | **frequency** |
| --- | --- | --- | --- |
| A | 1 | CTTTGTGTTTGGTCTGGGCA------------------------------------------CCAACATTATCACCACCC | 7x |
|  | 2 | CTTTGTGTTTGGTCTGGGC-------------------------TGT------------TCCCCAACATTATCACCACCC | 2x |
| H | 3 | CTTTGTGTTTGGTCTGGGC------------------------CTGT------------TCCCCAACATTATCACCACCC | 3x |
|  | 4 | CTTTGTGTTTGGTCTGGGCAC-----------------------TGT------------TCCCCAACATTATCACCACCC | 1x |
|  | 5 | CTTTGTGTTTGGTCTGGGCAC----------------------------------------CCCAACACTATCACCACCC | 1x |
| C | 6 | CTTTGTGTTTGGTCTGGGCACC------------------GGCCTGT------------TCCCCAACATTATCACCACCC | 2x |
|  | 7 | CTTTGTGTTTGGTCTGGGCACC----------------------TGT------------TCCCCAACATTATCACCACCC | 1x |
|  | 8 | CTTTGTGTTTGGTCTGGGCACCAGT-----------------CCTGT------------TCCCCAACATTATCACCACCC | 8x |
|  | 9 | CTTTGTGTTTGGTCTGGACACCAGTC-----------------CTGT------------TCCCCAACATTATCACCACCC | 1x |
|  | 10 | CTTTGTGTTTGGTCTGGGCACAAGTC-----------------CTGT------------TCCCCAACATTATCACCACCC | 1x |
|  | 11 | CTTTGTGTTTGGTCTGGGCACCAGTC-----------------C-GT------------TCCCCAACATTATCACCACCC | 2x |
| D | 12 | CTTTGTGTTTGGTCTGGGCACCAGTC--------------GGCCTGT------------TCCCCAACATTATCACCACCC | 12x |
| I | 13 | CTTTGTGTTTGGTCTGGGCACCA-TC--------------GGCCTGT------------TCCCCAACATTATCACCACCC | 5x |
| F | 14 | CTTTGTGTTTGGTCTGGGCACCAGAC--------------GGCCTGT------------TCCCCAACATTATCACCACCC | 1x |
|  | 15 | CTTTGTGTTTGGTCTGGGCACCAATC--------------GGCCTGT------------TCCCCAACATTATCACCACCC | 2x |
|  | 16 | CTTTGTGTTTGGTCTGGG------T-------------TTGGCCTGT------------TCCCCAACATTATCACCACCC | 1x |
| E | 17 | CTTTGTGTTTGGTCTGGGCACCAG-----------TTATCGGCCTGT------------TCCCCAACATTATCACCACCC | 2x |
|  | 18 | CTTTGTGTTTGGTCTGGGCACCAGTC-----------ATCGGCCAGT------------TCCCCAACATTATCACCACCC | 1x |
|  | 19 | CTTTGTGTTTGGTCTGGGCACCAGTC---------CAATCGGCCTGT------------TCCCCAACATTATCACCACCC | 1x |
|  | 20 | CTTTGTGTTTGGTCTGGGCACCAGTC---ccaacaTTATCGGCCTGT------------TCCCCAACATTATCACCACCC | 1x |
| J | 21 | CTTTGTGTTTGGTCTGGGCACCAGTC-----ggccTTATCGGCCTGT------------TCCCCAACATTATCACCACCC | 1x |
|  | 22 | CTTTGTGTTTGGTCTGGGCACCAGCC--tgtcggccTATCGGCCTGT------------TCCCCAACATTATCACCACCC | 2x |
|  | 23 | CTTTGTGTTTGGTCTGGGCACCAGACcaaacaggtGGTGGATAATGT------------TCCCCAACATTATCACCACCC | 1x |
|  | 24 | CTTTGTGTTTGGTCTGGGCACCAGTC-caggccagTTATCGGCCTGT------------TCCCCAACATTATCACCACCC | 1x |
|  | 25 | CTTTGTGTTTGGTCTGGGCACCAAACacaaa---GTGGTGATAATGT------------CCCCCAACATTATCACCACCC | 1x |
|  | 26 | CTTTGTGTTTGGTCTGGGCACCAGTCcggtgcccaGACTGGGCCTGTtccccaacattaTCCCCAACATTATCACCACCC | 1x |
|  |  | CTTTGTGTTTGGTCTGGGCACCAGTC---------TTATCGGCCTGT------------TCCCCAACATTATCACCACCC | 22x, wild-type |
|  |  |  | 84 clones |

Suppl. table 2d: Type and frequency of mutations in the neg slc45a2^#1^ fish (see Figure 1)

| **ID shared** | **#** | **Sequence** | **frequency** |
| --- | --- | --- | --- |
| B | 1 | CTTTGTGTT-------------------------------------------CGGCCTGTTCCCCAACATTATCACCACCC | 2x |
|  | 2 | CTTTGTGTTT-------------------------------------------GGCCTGTTCCCCAACATTATCACCACCC | 3x |
|  | 3 | CTTTGTGTTTGGT------------------------------------TATCGGCCTGTTCCCCAACATTATCACCACCC | 1x |
| D | 4 | CTTTGTGTCTGGTCTGGGCACCA------------------GT---------CGGCCTGTTCCCCAACATTATCACCACCC | 3x |
| J | 5 | CTTTGTGTTTGGTCTGGGCACCA------------------GTcggcCTTATCGGCCTGTTCCCCAACATTATCACCACCC | 1x |
|  | 6 | CTTTGTGTTTGGTCTGGGCACCAaactgggcaccactccccATgagcATTATCGGCCTGTTCCCCAACATTATCACCACCC | 2x |
| E | 7 | CTTTGTGTTTGGTCTGGGCACCA------------------GT------TATCGGCCTGTTCCCCAACATTATCACCACCC | 1x |
| A | 8 | CTTTGTGTTTGGTCTGGGCA-------------------------------------------CCAACATTATCACCACCC | 1x |
|  | 9 | CTTTGTGTTTGGTCTGGGCACCA------------------GT---------CGGACTGTTCCCCAACATTATCACCACCC | 1x |
| D | 10 | CTTTGTGTTTGGTCTGGGCACCA------------------GT---------CGGCCTGTTCCCCAACATTATCACCACCC | 8x |
| G | 11 | CTTTGTGTTTGGTCTGGGCACCA------------------GT-------ATCGGCCTGTTCCCCAACATTATCACCACCC | 1x |
|  | 12 | CTTTGTGTTTGGTCTGGGCACCA------------------GT----CTTATTGGCTTGTTCCCCAACATTATCACCACCC | 1x |
|  |  | CTTTGTGTTTGGTCTGGGCACCA------------------GT----CTTATCGGCCTGTTCCCCAACATTATCACCACCC | 63x, wild-type |
|  |  |  | 88 clones |

Suppl. table 2e:Type and frequency of mutations in the neg slc45a2^#2^ fish (see Figure 1).

| **ID shared** | **#** | **Sequence** | **frequency** |
| --- | --- | --- | --- |
|  | 1 | CTTTGTGTTAA---------------------------------------------TCGATATTATCACCACCC | 1x |
|  | 2 | CTTTGTGTTTGG------------------------------------CCTGTTCCCCAACATTATCACCACCC | 4x |
|  | 3 | CTTTGTGTTTGGT------------------------------------CTGTTCCCCAACATTATCGCCACCC | 2x |
| H | 4 | CTTTGTGTTTGGTCTGGG------------------------------CCTGTTCCCCAACATTATCACCACCC | 2x |
| A | 5 | CTTTGTGTATGGTCTGGGCA------------------------------------CCAACATTATCACCACCC | 1x |
|  | 6 | CTTTGTGTTTGGTCTGGGCACCAA------------------------------CCCCAACATTATCACCACCC | 1x |
| I | 7 | CTTTGTGTTTGGTCTGGGCACCA-TC--------------------GGCCTGTTCCCCAACATTATCACCACCC | 2x |
| D | 8 | CTTTGTGTTTGGTCTGGGCACCAGTC--------------------GGCCTGTTCCCCAACATTATCACCACCC | 12x |
|  | 9 | CTTTGTGTTTGGTCTGGGCACCAGTC-------------------CGGCCTGTTCCCCAACATTATCACCACCC | 1x |
|  | 10 | CTTTGTGTTTGGTCTGGGCACCAGTCGgcctgttccccaacaTATCGGCCTGTTCCCCAACATTATCACCACCC | 1x |
|  | 11 | CTTTGTGTTTGGTCTGGGCACCAGTC---------ggcctgtTATCGGCCTGTTCCCCAACATTATCACCACCC | 1x |
|  | 12 | CTTTGTGTTTGGTCTGGGCACCAGT-T-----atcggccagtTATCGGCCTGTTCCCTAACATTATCACCACCC | 1x |
| E | 13 | CTTTGTGTTTGGTCTGGGCACCAGT-----------------TATCGGCCTGTTCCCCAACATTATCACCACCC | 9x |
|  |  | CTTTGTGTTTGGTCTGGGCACCAGTCT---------------TATCGGCCTGTTCCCCAACATTATCACCACCC | 48x, wild-type |
|  |  |  | 86 clones |
