# Supplementary material for: Targeted Mutagenesis in Atlantic Salmon (Salmo salar L.) Using the CRISPR/Cas9 System Induces Complete Knockout Individuals in the F0 Generation
Source: PLoS One. 2014 Sep 25;9(9):e108622. doi: 10.1371/journal.pone.0108622 (PMC4177897; doi:10.1371/journal.pone.0108622)
Supplement: Table S3 — Indel types found in tyr fish presented in Figure 1 . (DOC) [file pone.0108622.s003.doc]

**Supplementary table S3.** Indels in CRISP*tyr*/Cas9 injected embryos. “ID shared” defines shared indel types for all 5 tested fish.

Suppl. table 3a: Type and frequency of mutations in the *tyr*-1 fish (see Figure 1)

| **ID shared** | **#** | **Sequence** | **frequency** |
| --- | --- | --- | --- |
| A | 1 | CTGCCCGGTGTGGGAGGGGGACGGGTCGG--------------------------------CCGAGGG | 1x |
|  | 2 | CTGCCCGGTGTGGGAGGAGGCCATGTCGG-------------------------------GCCGAGGG | 1x |
| B | 3 | CTGCCCGGTGTGGGAGGGGGACGGGTCGG-------------------------------GCCGAGGG | 10x |
|  | 4 | CTGCCCGGTGTGGGAGGGGGACGGGTC--------------------------ATGTCGGGCCGAGGG | 1x |
|  | 5 | CTGCCCGGTGTGGGAGGGGGACGCGGGGC-----------------------CATGTCGGGCCGAGGG | 1x |
| C | 6 | CTGCCCGGTGTGGGAGGGGGACGGGTCGG-----------------------CATGTCGGGCCGAGGG | 21x |
| D | 7 | CTGCCCGGTGTGGGAGGGGGACGGGTCGG----------------------CCATGTCGGGCCGAGGG | 2x |
| E | 8 | CTGCCCGGTGTGGGAGGGGGACGGGTGGG---------------------GCCATGTCGGGCCGAGGG | 3x |
|  | 9 | CTGCCCGGTGTGGGAGGGGGACGGGTCGG--------------------CAAAATGTCGGGCCGAGGG | 1x |
|  | 10 | CTGCCCGGTGTGGGAGGGGGACGGGTCGG------------ccatgtGGGGCCATGTCGGGCCGAGGG | 1x |
|  | 11 | CTGCCCGGTGTGGGAGGGGGACGGGTCGG-----------cacccgtGGGGCCATGTCGGGCCGAGGG | 1x |
|  | 12 | CTGCCCGGTGTGGGAGGGGGACGGGTCGGCAGCGcg--------agtGGGGCCATGTCGGGCCGAGGG | 2x |
|  | 13 | CTGCCCGGTGTGGGAGGGGGACGGGTCGGCATGTcgggccgacccgtGGGGCCATGTCGGGCCGAGGG | 12x |
|  |  | CTGCCCGGTGTGGGAGGGGGACGGGTCGGCATGT-------------GGGGCCATGTCGGGCCGAGGG | 1x, wild-type |
|  |  |  | 58 clones |

Target site

PAM

x insertion

X substitution

Suppl. table 3b: Type and frequency of mutations in the *tyr*-2 fish (see Figure 1)

| **ID shared** | **#** | **Sequence** | **frequency** |
| --- | --- | --- | --- |
|  | 1 | C-------------------------------------------TGTCGGGCCGAGGG | 1x |
|  | 2 | TGGCCATGT--------GGGG--------------------CCATGTCGGGCCGAGGG | 1x |
|  | 3 | CTGCCCGGTGTGGGGT-GGGG--------------------CCATGTCGGGCCGAGGG | 2x |
|  | 4 | CTGCCCGGTGTGGGAGtGGGG--------------------CCATGTCGGGCCGAGGG | 1x |
|  | 5 | CCGCCTGGTGTGGGAT-GGGG--------------------CCATGTCGGGCCGAGGG | 4x |
| F | 6 | CTGCCCGGTGTGGGAG-GGGG--------------------CCATGTCGGGCCGAGGG | 7x |
| B | 7 | CTGCCCGGTGTGGGAG-GGGGACG--------------------GGTCGGGCCGAGGG | 12x |
|  | 8 | CTGCCCGGTGTGGGAG-GGGGACGGGT--------------CCATGTCGGGCCGAGGG | 2x |
| G | 9 | CTGCCCGGTGTGGGAG-GGGGACGGGTCG------------CCATGTCGGGCCGAGGG | 2x |
| C | 10 | CTGCCCGGTGTGGGAG-GGGGACGGGTCGG------------CATGTCGGGCCGAGGG | 3x |
| E | 11 | CTGCCCGGTGTGGGAG-GGGGACGGGTGGG----------GCCATGTCGGGCCGAGGG | 3x |
| D | 12 | CTGCCCGGTGTGGGAG-GGGGACGGGTCGG-----------CCATGTCGGGCCGAGGG | 8x |
| H | 13 | CTGCCCGGTGTGGGAG-GGGGACGGGTCGG------TGGGGCCATGTCGGGCCGAGGG | 1x |
|  | 14 | CTGCCCGGTTTGGGAG-GGGGACGGGCCG-------TGGGGCCATGTCGGGCCGAGGG | 1x |
|  | 15 | CTGCCCGGTGTGGGAG-GGGGACGGGTCGGCA-------------------------- | 1x |
|  | 16 | CTGCCCGGTGTGGGAG-GGGGACGGGTCGGCAT----GGGGCCATGTCGGGCCGAGGG | 2x |
|  | 17 | CTGCCCGGTGTGGGAG-GGGGACGGGTCGGCCATggTGGGGCCATGTCGGGCCGAGGG | 1x |
|  | 18 | CTGCCCGGTGTGGGAG-GGGGACGGGTCGGGTGGggTGGGGCCATGTCGGGCCGAGGG | 1x |
|  |  | CTGCCCGGTGTGGGAG-GGGGACGGGTCGGCATG--TGGGGCCATGTCGGGCCGAGGG | 1x, wild-type |
|  |  |  | 54 clones |

Suppl. table 3c: Type and frequency of mutations in the *tyr*-3 fish (see Figure 1)

| **ID shared** | **#** | **Sequence** | **frequency** |
| --- | --- | --- | --- |
| F | 1 | CTGCCCGGTGTGGGAGGGGG-----------------------CCATGTC-------GGGCCGAGGG | 3x |
|  | 2 | CTGCCCGGTGTGGGAGGGGGAC----------------------CATGTC-------GGGCCGAGGG | 2x |
|  | 3 | CTGCCCGGTGTGGGAGGGGGCCATGTC-------------------------------GGCCGAGGG | 1x |
| A | 4 | CTGCCCGGTGTGGGAGGGGGACGGGTC-------------------------------GGCCGAGGG | 1x |
| B | 5 | CTGCCCGGTGTGGGAGGGGGACGGGTC------------------------------GGGCCGAGGG | 1x |
| G | 6 | CTGCCCGGTGTGGGAGGGGGACGGGTCG---------------CCATGTC-------GGGCCGAGGG | 6x |
| D | 7 | CTGCCCGGCGTGGGAGGGGGACGGGTCGG--------------CCATGTC-------GGGCCGAGGG | 1x |
|  | 8 | CTGCCCGGTGTGGGAGGGGGACGGGTCGG---------------CAT---------------GAGGG | 1x |
| C | 9 | CTGCCCGGTGTGGGAGGGGGACGGGTCGG---------------CATGTC-------GGGCCGAGGG | 5x |
| E | 10 | CTGCCCGGTGTGGGAGGGGGACGGGTGGG-------------GCCATGTC-------GGGCCGAGGG | 6x |
|  | 11 | CTGCCCGGTGTGGGAGGGGGACGGGTCGG-----------TGGCGCTGTC-------GGGCCGAGGG | 2x |
| H | 12 | CTGCCCGGTGTGGGAGGGGGACGGGTCGG---------TGGGGCCATGTC-------GGGCCGAGGG | 1x |
|  | 13 | CTGCCCGGTGTGGGAGGGGGACGGGCCGG-A-GT-----GGGGCCATGTC-------GGGCCGAGGG | 1x |
|  | 14 | CTGCCCGGTGTGGGAGGGGGACGGGTCGGCAGCAtctgtGGGGCCATGTC-------GGGCCGAGGG | 1x |
|  | 15 | CTGCCCGGTGTGGGAGGGGGACGGGTCGGCGGGT-----GGGGCCATGTCgggccgaGGGCCGAGGG | 1x |
|  | 16 | CTGCCCGGTGTGGGAGGGGGACGGGTCGGCATGT-----CGGGCCATGTC-------GGGCCGAGGG | 1x |
|  | 17 | CTGCCCGGTGTGGGAGGAGGACGGGTCGGCATGT-----GGGGCCATGTC-------GGGCCGAGGG | 1x |
|  |  | CTGCCCGGTGTGGGAGGGGGACGGGTCGGCATGT-----GGGGCCATGTC-------GGGCCGAGGG | 12x, wild-type |
|  |  |  | 47 clones |

Suppl. table 3d: Type and frequency of mutations in the neg *tyr*^#1^ fish (see Figure 1)

| **ID shared** | **#** | **Sequence** | **frequency** |
| --- | --- | --- | --- |
|  | 1 | CTGCCCGGTGTGGGAGGGGGACGGGTCGGCACGTGGGGCCATGTCGGGCCGAGGG | 1x |
|  | 2 | CTGCCCGGTGTGGGAGGGGGACGGGTCAGCATGTGGGGCCATGTCGGGCCGAGGG | 3x |
|  |  | CTGCCCGGTGTGGGAGGGGGACGGGTCGGCATGTGGGGCCATGTCGGGCCGAGGG | 46x, wild-type |
|  |  |  | 50 clones |

Suppl. table 3e: Type and frequency of mutations in the neg *tyr*^#2^ fish (see Figure 1)

| **ID shared** | **#** | **Sequence** | **frequency** |
| --- | --- | --- | --- |
|  | 1 | CTGCCCGGTGTGGGAGGGGGGCGGGTCGGCATGTGGGGCCATGTTGGGCCGAGGG | 1x |
|  | 2 | CTGCCCGGTGTGGGAGGGGGACGGATCGGCATGTGGGGCCATGTCGGGCCGAGGG | 1x |
|  | 3 | CTGCCCGGTGTGGGAGGGGGACGGGTCGGCGTGTGGGGCCATGTCGGGCCGAGGG | 1x |
|  |  | CTGCCCGGTGTGGGAGGGGGACGGGTCGGCATGTGGGGCCATGTCGGGCCGAGGG | 39x, wild-type |
|  |  |  | 42 clones |
