# Supplementary material for: Targeted Mutagenesis in Atlantic Salmon (Salmo salar L.) Using the CRISPR/Cas9 System Induces Complete Knockout Individuals in the F0 Generation
Source: PLoS One. 2014 Sep 25;9(9):e108622. doi: 10.1371/journal.pone.0108622 (PMC4177897; doi:10.1371/journal.pone.0108622)
Supplement: Table S4 — Indel types found in slc45a2 fish presented in Figure 2 . (DOC) [file pone.0108622.s004.doc]

**Supplementary Table S4.** Indel types and frequencies of CRISP*slc45a2*/Cas9 injected embryos in the *slc45a2*-4, *slc45a2*-5 and *slc45a2*-6 whole embryo vs. fin clip samples (see Figure 2). “ID shared” defines shared indel types for all 3 tested fish.

Suppl. table 4a: Indels found in the *slc45a2*-4 fish a comparison between whole embryo and fin clip (Figure 2).

| **ID shared** | **sequence** | **Frequency of indel types** | |
| --- | --- | --- | --- |
|  |  | **Whole fish** | **Fin clip** |
| A | TGTTTGGTCTGGGCACCAGT-----------CGGCCTGTTCCCCAACATTATCACCACCCTCATCCTGTGCA | 21 | 2 |
|  | TGTTTGGTCTGGGCACCAGTCtg----TTATCGGCCTGTTCCCCAACATTATCACCACCCTCATCCTGTGCA | 21 | 0 |
|  | TGTTTGGTCTGGGCACCAGTGcgtctcCTATCGGCCTGTTCCCCAACATTATCACCACCCTCATCCTGTGCA | 12 | 3 |
|  | TGTTTGGTCTGGGCACCAGT--------------CCTGTTCCCCAACATTATCACCACCCTCATCCTGTGCA | 11 | 5 |
| C | TGTTTGG---------------------------CCTGTTCCCCAACATTATCACCACCCTCATCCTGTGCA | 11 | 4 |
|  | TGTTTGGTCTGGGCACGATAGaca-----ATCGGCCTGTTCCCCAACATTATCACCACCCTCATCCTGTGCA | 3 | 2 |
|  | TGTTTGGTCTGGGCACCAGTC-caacaACATCGGCCTGTTCCCCAACATTATCACCACCCTCATCCTGTGCA | 2 | 0 |
|  | TGTTTGGTCTGGGCACCAGTGcgtctcCTATCGACCTGTTCCCCAACATTATCACCACCCTCATCCTGTGCA | 1 | 0 |
|  | TGTTTGGTCTGGGCA-------------------CCTGTTCCCCAACATTATCACCACCCTCATCCTGTGCA | 1 | 0 |
|  | TGTTTGGTCTGGGCACCAGA---------------------CCCAACATTATCACCACCCTCATCCTGTGCA | 1 | 0 |
|  | TGTTTGGTCTGGGCACCTGT-----------CGGCCTGTTCCCCAACATTATCACCACCCTCATCCTGTGCA | 1 | 0 |
|  | TGTTTGGTCTGGGCACCAGTC----tgTTATCGACCTGTTCCCCAACATTATCACCACCCTCATCCTGTGCA | 0 | 3 |
| G | TGTTTGGTCTGGGCACCAGT--------TATCGGCCTGTTCCCCAACATTATCACCACCCTCATCCTGTGCA | 0 | 1 |
| B | TGTTTGG---------------------------TCTGTTCCCCAACATTATCACCACCCTCATCCTGTGCA | 0 | 1 |

PAM

Suppl. table 4b: Indels found in the *slc45a2*-5 fish a comparison between whole embryo and fin clip (Figure 2).

| **ID shared** | **sequence** | **Frequency of indel types** | |
| --- | --- | --- | --- |
|  |  | **Whole fish** | **Fin clip** |
| A | TGTTTGGTCTGGGCACCAGT-------------CGGCCTGTTCCCCAACATTATCACCACCCTCATCCTGTGCA | 48 | 11 |
|  | TGTTTGGTCTGGGCACCAGCTGGGT-------CCGGCCTGTTCCCCAACATTATCACCACCCTCATCCTGGGCA | 9 | 3 |
|  | TGTTTGGTCTGGGAACCAGT----------------------------------CACCACCCTCATCCTGTGCA | 8 | 0 |
| F | TGTTTGGTCTGGGCACCAGA------------CCGGCCTGTTCCCCAACATTATCACCACCCTCATCCTGTGCA | 6 | 0 |
|  | TGTTTGGTCTGAGATTTA--------------TCGGCCTGTTCCCCAACATTATCACCACCCTCATCCTGGGCA | 4 | 2 |
|  | TGTTTGGTCTGGGCACCAG---------------------------------------ACCCTCATCCTGTGCA | 1 | 2 |
|  | TGTTTGGTCTGGGCACCAGTCATTGTCTGTTATCGGCCTGTTCCCCAACATTATCACCACCCTCATCCTGTGCA | 1 | 0 |
| E | TGTTTGGTCTGGGCACCAGTC--------TTATCGGCCTGTTCCCCAACATTATCACCACCCTCATCCTGTGCA | 1 | 0 |
| G | TGTTTGGTCTGGGCACCAGT----------TATCGGCCTGTTCCCCAACATTATCACCACCCTCATCCTGTGCA | 1 | 0 |
|  | TGT-----------------------------TCGGCCTGCTCCCCAACATTATCACCACCCTCATCCTGTGCA | 1 | 0 |
|  | TGTTTGGTCTGGGCACCAAACTGT--------TCGGCCTGTTCCCCAACATTATCACCACCCTCATCCTGTGCA | 1 | 0 |
|  | TGTTTGGTCTGGGCACCAG-------------CCGGCCTGTTCCCCAACATTATCACCACCCTCATCCTGTGCA | 1 | 0 |
|  | TGTTTGGTCTGGGCA-----------------CCGGCCTGTTCCCCAACATTATCACCACCCTCATCCTGTGCA | 1 | 0 |
|  | TGTTTGGTCTGGGCACCAAACACA--------AAGGCCTGTTCCCCAACATTATCACCACCCTCATCCTGTGCA | 1 | 0 |
|  | TGTTTGGTCTGGGCACCAGT----------------------------------------CCGCATT---TGCA | 1 | 0 |
| D | TGTTTGGTCTGGGCACCA-----------------------------ACATTATCACCACCCTCATCCTGTGCA | 1 | 0 |
|  | TGTTTGGTCTGGGCACCAG----------------------------------TCACCACCCTCATCCTGTGCA | 0 | 2 |

Suppl. table 4c: Indels found in the *slc45a2*-6 fish a comparison between whole embryo and fin clip (Figure 2).

| **ID shared** | **Sequence** | **Frequency of indel types** | |
| --- | --- | --- | --- |
|  |  | **Whole fish** | **Fin clip** |
| A | TGTTTGGTCTGGGCACCAG-------------------------------------TCACCACCCTCATCCTGTGCA | 8 | 2 |
|  | TGTTTGGTCTGGGCACCA-------------------GGCCTGTTCCCCAACATTATCACCACCCTCATCCTGTGCA | 7 | 0 |
| D | TGTTTGGTCTGGGCA--------------------------------CCAACATTATCACCACCCTCATCCTGTGCA | 5 | 3 |
|  | TGTTTGGTCTGGGCACC--------------------GGCCTGTTCCCCAACATTATCACCACCCTCATCCTGTGCA | 5 | 1 |
| E | TGTTTGGTCTGGGCACCAGTC-----------TTATCGGCCTGTTCCCCAACATTATCACCACCCTCATCCTGTGCA | 4 | 7 |
|  | TGTTTGGTCTGGGCACCA--------------------GCCTGTTCCCCAACATTATCACCACCCTCATCCTGTGCA | 2 | 0 |
|  | TGTTTGGTCTGGGCACCAGTC-------GGCCTTATCGGCCTGTTCCCCAACATTATCACCACCCTCATCCTGTGCA | 2 | 0 |
|  | TGTTTGGTCTGGGCACCAGTC-------GGCCTTATCGGCCTGTTCCCCAACATTATCACCACCCTCATCCTGTGCA | 2 | 0 |
|  | TGTTTGGTCTGGGCACCAAACACAAAGTATCCTTATCGGCCTGTTCCCCAACATTATCACCACCCTCATCCTGTGCA | 1 | 3 |
|  | TGTTTGGTCTGGGCACCAGTT-------------ATCGGCCTGTTCCCCAACATTATCACCACCCTCATCCTGTGCA | 1 | 0 |
|  | TGTTTGGTCTCGGC--------------------------CTGTTCCCCAACATTATCACCACCCTCATCCTGTGCA | 1 | 0 |
|  | TGTTTGGTCTGGGCACCAG---------------------------------------------------------- | 1 | 0 |
|  | TGTTTGGTCTGGGCACCAG-------------------------------ACATTATCACCACCCTCATCCTGTGCA | 1 | 0 |
| F | TGTTTGGTCTGGGCACCAGAC---------------CGGCCTGTTCCCCAACATTATCACCACCCTCATCCTGTGCA | 1 | 0 |
|  | TGTTTGGTCTGGGCACCA-----------------CCGGCCTGTTCCCCAACATTATCACCACCCTCATCCTGTGCA | 1 | 0 |
|  | -----------------------------------------------------TTATCGGCCTGTTCCCCAACATTA | 1 | 0 |
|  | TGTTTGGTCTGGGCACC---------------TTATCGGCCTGTTCCCCAACATTATCACCACCCTCATCCTGTGCA | 1 | 0 |
|  | TGTTTGGTCTGGGCACCAGTC----------TGTATCGGCCTGTTCCCCAACATTATCACCACCCTCATCCTGTGCA | 1 | 0 |
|  | TGTTTGGTCTGGGCACCAGGA----------CGCACTGGCCTGTTCCCCAACATTATCACCACCCTCATCCTGTGCA | 1 | 0 |
|  | TGTTTGGTCTGGGCACCAGTC---------CAGTATCGGCCTGTTCCCCAACATTATCACCACCCTCATCCTGTGCA | 1 | 0 |
|  | TGTTTGGTCTGGGCACCAGTC-------GGCCTGTTCGGCCTGTTCCCCAACATTATCACCACCCTCATCCTGTGCA | 1 | 0 |
|  | TGTTTGGTCTGGGCACCAGAC-----GGCCTATTATCGGCCTGTTCCCCAACATTATCACCACCCTCATCCTGTGCA | 1 | 0 |
|  | TGTTTGGTCTGGGCACCAGGC------ACCAGTTATCGGGCTGTTCCCCAACATTATCACCACCCTCATCCTGTGCA | 1 | 0 |
|  | TGTTTGGTCTGGGCACCAGTC-TGGGCACCAGTTATCGGCCTGTTCCCCAACATTATCACCACCCTCATCCTGTGCA | 1 | 0 |
| B | TGTTTGGT--------------------------------CTGTTCCCCAACATTATCACCACCCTCATCCTGTGCA | 0 | 2 |
|  | TGTGTGTT--------------------------ATCGGCCTGTTCCCCAACATTATCACCACCCTCATCCTGTGCA | 0 | 2 |
| C | TGTTTG--------------------------------GCCTGTTCCCCAACATTATCACCACCCTCATCCTGTGCA | 0 | 1 |
|  | TGTTTGGTCTGGGCACCAG-------------TT-CCGGCCTGTTCCCCAACATTATCACCACCCTCATCCTGTGCA | 0 | 1 |
